# Occupational Class and Exposure to Job Stressors among Employed Men and Women in Japan

**DOI:** 10.2188/jea.14.204

**Published:** 2005-03-18

**Authors:** Norito Kawakami, Takashi Haratani, Fumio Kobayashi, Masao Ishizaki, Takeshi Hayashi, Osamu Fujita, Yoshiharu Aizawa, Shogo Miyazaki, Hisanori Hiro, Takeshi Masumoto, Shuji Hashimoto, Shunichi Araki

**Affiliations:** 1 Hygiene and Preventive Medicine, Okayama University Graduate School of Medicine and Dentistry.; 2 National Institute of Industrial Health.; 3 Aichi Medical University.; 4 Kanazawa Medical University.; 5 Hitachi Information & Telecomunication Systems, Ltd.; 6 Aichi Education University.; 7 Kitasato University School of Medicine.; 8 Meiji University Law School.; 9 Adecco Health Support Center.; 10 Mitsubishi Chemical Corporation.; 11 Fujita Health University School of Medicine.

**Keywords:** occupational groups, job demands, job control, job insecurity, worksite social support

## Abstract

BACKGROUND: The relationship between occupational class and exposure to job stressors among employed men and women in Japan remains unclear.

METHODS: Data of 16,444 men and 3,078 women were analyzed. The information was obtained from answers to a questionnaire distributed among employees of nine companies in Japan between 1996 and 1998 (average response rate, 85%). The International Standardized Classification of Occupations was used to classify respondents into eight occupational categories. The Job Content Questionnaire was used to measure job demands, job control, worksite support, and job insecurity. The associations between occupational class and job stressors, as well as job strain, were examined controlling for age, education, marital status, chronic medical condition, and personality traits, such as neuroticism and extraversion.

RESULTS: Men and women in high-class occupations (e.g., managers and professionals) had significantly greater job control, while job demands and worksite social support were not greatly different among occupations. A clear occupational class gradient in job insecurity was observed in women. A greater prevalence of high job strain was observed in low-class occupations compared to high-class occupations in both men and women. The occupational class gradient in job strain was greater for women. These patterns did not change after controlling for other covariates.

CONCLUSION: The present study suggests an occupational class gradient in job strain for employed men and women in Japan. Japanese women workers may have a greater occupational class gradient in job strain and job insecurity than men.

A clear gradient of health status has been observed across employment grades or occupational classes.^1^ One possible explanation of the occupational-class gradient of health status among workers is the psychosocial work environment, particularly job strain^2^ defined as the combination of greater job demands and lower job control (i.e., decision-latitude).^1^ A recent cross-national comparison of perceived health and physical functioning in men and women civil servants in Japan, the United Kingdom, and Finland found that, for men, the association between employment grade and health status was weaker in Japan than in the other two countries.^3^ For women, the association was inconsistent between the two survey sites in Japan, and almost no association was observed at one of the sites.^3^ Similarly, in contrast to previous Western findings, greater coronary heart disease risk factors^4^ and lower leisure-time physical activity were observed among men in high-class occupations in Japan.^5^ A weaker or inconsistent association between occupational class and health status in these Japanese samples may come from a less-clear association between occupational class and exposure to adverse psychosocial factors at work, such as job strain.

Research on occupational class and exposure to job stressors is limited in Japan. It has been reported that job control was greater and job strain was lower in managers and white-collar workers than in blue-collar workers or vehicle operators among Japanese men,^6^^-^^8^ consistently with previous observations in Western countries,^1^^,^^9^ while the patterns for social support at work were inconsistent.^6^^,^^8^ However, in most previous studies, only dichotomous categories of occupation, such as managers vs. other workers, or white-collar vs. blue-collar workers, were used.^6^^-^^8^ Among women, research on this topic is quite limited in Japan. A previous study^7^ found greater job strain among blue-collar workers than among white-collar workers. Another study indicated that the relationship between occupation and job stressors was less clear among women employees of a computer company.^10^

The objective of the present study is to clarify the occupational class gradient of exposure to job stressors in Japanese men and women with a greater variety of occupational categories and a large sample of workers from multiple companies/organizations. We employed the 1988 version of the International Standardized Classification of Occupations (ISCO88)^11^ and an internationally standardized self-report measure of psychosocial job stressors (JCQ).^12^ We examined the association controlling for personality traits (neuroticism and extraversion) which have been shown to have an effect on the reporting of job strain.^13^

## METHODS

### Subjects

Nine companies or factories located in the Kanto (east coast) and Chubu (central Japan) regions were selected by collaborators and agreed to participate in the study. They included a light-metal fabricator, three electrical manufacturers, two steel manufacturers, a heavy-metal manufacturer, an automobile company, and a car parts producer. Only full-time employees were invited to participate in the study. At four study sites, all full-time employees were invited. At three other sites, full-time employees who participated in health checkups within a certain period were invited. At one site, full-time employed men who had participated in health checkups were invited. At another site, all supervisors and managers were invited. A questionnaire was distributed by mail with a letter of invitation explaining the objectives and procedure of the study to a total of 29,417 eligible subjects between April 1996 and May 1998. The subjects were asked to complete the questionnaire at the worksite or at home and return it in a thick envelope with their written consent to participate to occupational health division. Information on their IDs was also collected for a future linkage with medical records. A total of 25,104 questionnaires were returned. The average response rate was 85%, ranging from 73% to 100% at most study sites, with an exception (43%) at one site. We excluded 3,026 responses from one study site collected during the health checkups between June 1997 and November 1997 because the questionnaire that was distributed during that period lacked a part of the JCQ scales due to an editorial mistake. In addition, 2,421 respondents were excluded because of at least one missing response for variables relevant to the study. Furthermore, a small number of respondents (n=135) who reported their occupation as farming or in a miscellaneous category were excluded from the following analyses. The data from 19,522 respondents (16,444 men and 3,078 women) were analyzed.

### Classification of occupation

The ISCO88 classifies and ranks occupations according to the levels of skills required and education needed to perform a particular occupation.^11^ Legislators and managers were ranked the highest, followed by professionals, technicians, clerks, service and sales workers, craft and related trade workers, and machine operators and assemblers. Laborers were ranked the lowest. Respondents were asked to briefly describe their job titles and their most important roles at work, as well as to select their occupation from a multiple-choice question. Based on the descriptions, a four-digit occupational code was entered from the ISCO88 by trained raters under the supervision of researchers (TH, NK). In this study, the first digit of the ISCO88 occupation code (except for military and agricultural/fishery occupations) was used to determine eight occupation categories: managers, professionals, technicians, clerks, service and sales workers, craft and related trade workers, machine operators and assemblers, and laborers. A thorough review of the respondent’s description and coding revealed a problem with this procedure: a number of respondents who rated themselves as having managerial occupations (a section chief or a higher position) in the multiple-choice question regarding occupation did not mention their positions as managers in the description section. Thus, their occupations were assigned as “managers” when the respondents selected “managers” in the multiple-choice question. A total of 430 respondents (2% of the total) returned a blank response to the question requiring a description of their occupation. The occupations of these respondents were classified based on their response to the multiple-choice question. As noted above, a small number of respondents (n=135) who reported their occupation as agricultural or miscellaneous or who did not report any information on occupation were excluded from the analyses.

### Assessment of job stressors

The questionnaire included the following JCQ scales to assess job stressors: five-item psychological job demands scale, nine-item job control (job decision-latitude) scale, four-item supervisor support scale, four-item coworker support scale, and four-item job insecurity scale.^12^^,^^14^ Each question was asked using 4 response options and item scores were weighted and summed up to calculate a scale score. High scale scores (the range of score) indicate a greater quantitative workload (12-48), greater learning opportunity and influence at work (24-96), greater social support from supervisors (4-16) and from coworkers (4-16) in the workplace, and greater job insecurity (4-17), respectively. The Japanese version of these scales has shown acceptable levels of reliability and validity in previous studies.^15^^,^^16^ Cronbach’s alpha reliability coefficients for the scales ranged from 0.61 to 0.89 for men and from 0.65 to 0.87 for women. In addition, the scales showed factor-based validity, with distributions of the scale scores across age groups and occupations being in the expected direction.^15^ Operationally, a group with a high degree of job strain was defined as follows. First, the respondents were dichotomized in terms of scores for job demands or job control using an average for each gender group. Respondents with high job demands and low job control were defined as the group with high job strain.

### Other covariates

Other covariates consisted of age, education, marital status, chronic medical condition, personality traits, and survey site. Age was classified into three groups, 18-34 years old, 35-44 years old, and 45 years old or older. Education was classified into three groups, i.e., less than high school graduates, high school graduates, and college graduates or higher. Marital status was classified into three groups: currently married, never married, and previously married. The subjects were categorized as having a chronic medical condition (any circulatory disease, cancer, gastrointestinal disease, or muskeloskeletal problems) for which they were currently receiving medical treatment. Neuroticism and extraversion were measured using scales from the short version of Eysenck’s Personality Questionnaire Revised (SEPQ-R).^17^ Each scale consisted of 12 items with a yes/no response yielding a total score of 0-12; a high score reflects the degree of nervousness or anxiety experienced by the respondent for neuroticism or the degree of participation and interaction with others in social situations for extroversion. The Japanese version of SEPQ-R was proven to be reliable and valid.^18^ Respondents were classified according to their scores into three groups of equal sizes, i.e., high, medium, and low, depending on the degree of each personality trait expressed.

### Statistical analysis

The associations of occupational class and job stressors were examined separately by gender. First, average scores of the five job stressors were compared among the eight occupational categories using one-way analysis of variance (ANOVA) or multivariate ANOVA controlling for the covariates to test the significant difference. The linear trend was also examined. The percent of variance of each job stressor score explained by occupation was calculated as the ratio of the sum-of-the-square for occupation to that for residuals in a multivariate ANOVA. Second, the proportion of those in the high job strain group was compared among occupations (Chi-square test). A multiple logistic regression analysis of the high job strain group was conducted on occupation controlling for the covariates. The estimated prevalence odds ratios (ORs) of the high job strain group and its 95% confidence intervals (CIs) were calculated taking managers as a reference group for men and a combined category of managers and professionals as a reference for women because of the small number of female managers. The p value of significance was set as 0.05 or less. These analyses were conducted using a statistical package, SPSS^®^ 11.0J (SPSS, Inc., Chicago, IL, USA).

### Ethics

The Human Subjects Committee of Gifu University School of Medicine, Japan, approved the recruitment, consent, and field procedures before the survey was conducted.

## RESULTS

Table 1 shows the distribution of the covariates by occupations. The averages and standard deviations of job stressor scores were 32.8 (5.2) for job demands, 67.4 (10.9) for job control, 10.8 (2.2) for supervisor support, 11.2 (1.6) for coworker support, and 6.7 (1.7) for job insecurity among men. Those values were 31.4 (5.2) for job demands, 58.9 (10.4) for job control, 10.5 (2.4) for supervisor support, 11.0 (1.7) for coworker support, and 7.0 (2.0) for job insecurity among women.

**Table 1.** Demographic variables, chronic medical condition, study site (company/worksite) and personality traits by occupational class.

	Managers	Professionals	Technicians	Clerks	Service & sales workers	Craft & related trade workers	Machine operators and assemblers	Laborers	Gender total
								
	n (%)	n (%)	n (%)	n (%)	n (%)	n (%)	n (%)	n (%)	n (%)
					men		men		
	(n=2762)	(n=2547)	(n=2370)	(n=1371)	(n=230)	(n=1864)	(n=4921)	(n=379)	(n=16444)
age (year)**									
18-34	3 (0.1)	567 (22.3)	789 (33.3)	288 (21.0)	18 (7.8)	396 (21.2)	1,499 (30.5)	59 (15.6)	3,619 (22.0)
35-44	1,157 (41.9)	1,564 (61.4)	1,025 (43.2)	548 (40.0)	93 (40.4)	651 (34.9)	1,551 (31.5)	92 (24.3)	6,681 (40.6)
45 or older	1,602 (58.0)	416 (16.3)	556 (23.5)	535 (39.0)	119 (51.7)	817 (43.8)	1,871 (38.0)	228 (60.2)	6,144 (37.4)
marital status**									
married	2,643 (95.7)	1,988 (78.1)	1,749 (73.8)	1,093 (79.7)	194 (84.3)	1,441 (77.3)	3,558 (72.3)	312 (82.3)	12,978 (78.9)
never married	85 (3.1)	530 (20.8)	567 (23.9)	259 (18.9)	27 (11.7)	386 (20.7)	1,255 (25.5)	59 (15.6)	3,168 (19.3)
previously married	34 (1.2)	29 (1.1)	54 (2.3)	19 (1.4)	9 (3.9)	37 (2.0)	108 (2.2)	8 (2.1)	298 (1.8)
education**									
less than high school	44 (1.6)	30 (1.2)	87 (3.7)	88 (6.4)	43 (18.7)	311 (16.7)	975 (19.8)	107 (28.2)	1,685 (10.2)
high school or some college	821 (29.7)	1,039 (40.8)	1,281 (54.1)	880 (64.2)	144 (62.6)	1,469 (78.8)	3,843 (78.1)	258 (68.1)	9,735 (59.2)
college or higher	1,897 (68.7)	1,478 (58.0)	1,002 (42.3)	403 (29.4)	43 (18.7)	84 (4.5)	103 (2.1)	14 (3.7)	5,024 (30.6)
chronic medical condition**									
none	2,496 (90.4)	2,378 (93.4)	2,198 (92.7)	1,252 (91.3)	195 (84.8)	1,686 (90.5)	4,492 (91.3)	330 (87.1)	15,027 (91.4)
any	266 (9.6)	169 (6.6)	172 (7.3)	119 (8.7)	35 (15.2)	178 (9.5)	429 (8.7)	49 (12.9)	1,417 (8.6)
company/worksite**									
A	450 (16.3)	314 (12.3)	534 (22.5)	331 (24.1)	39 (17.0)	742 (39.8)	1,707 (34.7)	244 (64.4)	4,361 (26.5)
B	336 (12.2)	133 (5.2)	135 (5.7)	31 (2.3)	16 (7.0)	76 (4.1)	75 (1.5)	9 (2.4)	811 (4.9)
C	39 (1.4)	30 (1.2)	106 (4.5)	51 (3.7)	5 (2.2)	268 (14.4)	443 (9.0)	12 (3.2)	954 (5.8)
D	1,357 (49.1)	1,505 (59.1)	806 (34.0)	581 (42.4)	100 (43.5)	454 (24.4)	1,293 (26.3)	82 (21.6)	6,178 (37.6)
E	318 (11.5)	106 (4.2)	56 (2.4)	52 (3.8)	18 (7.8)	5 (0.3)	3 (0.1)	0	558 (3.4)
F	38 (1.4)	27 (1.1)	102 (4.3)	31 (2.3)	13 (5.7)	84 (4.5)	226 (4.6)	10 (2.6)	531 (3.2)
G	199 (7.2)	348 (13.7)	442 (18.6)	168 (12.3)	8 (3.5)	97 (5.2)	534 (10.9)	4 (1.1)	1,800 (10.9)
H	7 (0.3)	65 (2.6)	128 (5.4)	47 (3.4)	22 (9.6)	74 (4.0)	250 (5.1)	8 (2.1)	601 (3.7)
I	18 (0.7)	19 (0.7)	61 (2.6)	79 (5.8)	9 (3.9)	64 (3.4)	390 (7.9)	10 (2.6)	650 (4.0)
neuroticism*^, †^	[ 4.9 (3.2) ]	[ 5.4 (3.2) ]	[ 5.5 (3.3) ]	[ 5.2 (3.3) ]	[ 4.8 (3.2) ]	[ 5.2 (3.2) ]	[ 5.5 (3.3) ]	[ 5.1 (3.3) ]	[ 5.3 (3.3) ]
extraversion**^, †^	[ 6.3 (3.6) ]	[ 5.0 (3.5) ]	[ 5.2 (3.5) ]	[ 5.6 (3.7) ]	[ 6.1 (3.6) ]	[ 5.4 (3.5) ]	[ 5.3 (3.5) ]	[ 5.8 (3.7) ]	[ 5.5 (3.6) ]

	women								
	(n=9)	(n=97)	(n=153)	(n=1013)	(n=102)	(n=155)	(n=1248)	(n=301)	(n=3078)
age (year)**									
18-34	5 (56)	78 (80.4)	102 (66.7)	698 (68.9)	11 (10.8)	34 (21.9)	456 (36.5)	45 (15.0)	1,429 (46.4)
35-44	3 (33)	13 (13.4)	43 (28.1)	197 (19.4)	24 (23.5)	56 (36.1)	346 (27.7)	86 (28.6)	768 (25.0)
45 or older	1 (11)	6 (6.2)	8 (5.2)	118 (11.6)	67 (65.7)	65 (41.9)	446 (35.7)	170 (56.5)	881 (28.6)
marital status**									
married	5 (56)	46 (47.4)	90 (58.8)	542 (53.5)	88 (86.3)	128 (82.6)	914 (73.2)	248 (82.4)	2,061 (67.0)
never married	4 (44)	49 (50.5)	59 (38.6)	450 (44.4)	3 (2.9)	17 (11.0)	274 (22.0)	31 (10.3)	887 (28.8)
previously married	0	2 (2.1)	4 (2.6)	21 (2.1)	11 (10.8)	10 (6.5)	60 (4.8)	22 (7.3)	130 (4.2)
education**									
less than high school	0	1 (1.0)	4 (2.6)	21 (2.1)	49 (48.0)	56 (36.1)	359 (28.8)	136 (45.2)	626 (20.3)
high school or some college	9 (100)	73 (75.3)	132 (86.3)	938 (92.6)	50 (49.0)	98 (63.2)	888 (71.2)	162 (53.8)	2,350 (76.3)
college or higher	0	23 (23.7)	17 (11.1)	54 (5.3)	3 (2.9)	1 (0.6)	1 (0.1)	3 (1.0)	102 (3.3)
chronic medical condition**									
none	7 (78)	94 (96.9)	146 (95.4)	983 (97.0)	95 (93.1)	147 (94.8)	1,172 (93.9)	277 (92.0)	2,921 (94.9)
any	2 (22)	3 (3.1)	7 (4.6)	30 (3.0)	7 (6.9)	8 (5.2)	76 (6.1)	24 (8.0)	157 (5.1)
company/worksite**									
A	4 (44)	60 (61.9)	110 (71.9)	682 (67.3)	100 (98.0)	148 (95.5)	1,086 (87.0)	276 (91.7)	2,466 (80.1)
B	1 (11)	0	0	34 (3.4)	0	0	0	0	35 (1.1)
C	0	5 (5.2)	15 (9.8)	14 (1.4)	0	2 (1.3)	1 (0.1)	3 (1.0)	40 (1.3)
D	0	2 (2.1)	1 (0.7)	1 (0.1)	0	0	0	0	4 (0.1)
F	1 (11)	2 (2.1)	4 (2.6)	47 (4.6)	0	0	4 (0.3)	7 (2.3)	65 (2.1)
G	3 (33)	25 (25.8)	17 (11.1)	173 (17.1)	1 (1.0)	5 (3.2)	149 (11.9)	15 (5.0)	388 (12.6)
H	0	3 (3.1)	5 (3.3)	52 (5.1)	1 (1.0)	0	8 (0.6)	0	69 (2.2)
I	0	0	1 (0.7)	10 (1.0)	0	0	0	0	11 (0.4)
neuroticism*^, †^	[ 5.6 (3.2) ]	[ 5.6 (3.3) ]	[ 5.9 (2.9) ]	[ 6.1 (3.1) ]	[ 4.9 (3.1) ]	[ 6.0 (3.2) ]	[ 6.0 (3.2) ]	[ 5.9 (3.4) ]	[ 6.0 (3.2) ]
extraversion**^, †^	[ 6.2 (3.7) ]	[ 6.5 (3.4) ]	[ 5.9 (3.2) ]	[ 6.1 (3.4) ]	[ 6.1 (3.5) ]	[ 5.4 (3.3) ]	[ 5.6 (3.4) ]	[ 5.7 (3.4) ]	[ 5.8 (3.4) ]

* p<0.05, ** p<0.01 (Chi-square test or one-way ANOVA).

† : Average with standard deviation in parentheses is shown.

For men and women, the scores for five job stressors were significantly different among occupational categories (Table 2). Those in high-class occupations (such as managers and professionals) reported greater job control than those in low-class occupations (such as machine operators and laborers) in men and women. To a lesser extent, those in high-class occupations had greater scores of job demands, supervisor support, and coworker support and a lower score of job insecurity than those in low-class occupations. The differences in supervisor support and job insecurity were slightly greater in women than in men. The patterns did not change after controlling for the covariates, while the linear trend for job demands was no more significant in women. The proportion of variance explained by occupations using multivariate ANOVA was 12.1% and 13.1% for job control in men and women, respectively; 2.0% for supervisor support in women; 5.0% for job insecurity in women; and, otherwise, 0.4-0.8%.

**Table 2.** Crude average (standard deviation, SD) and multivariate adjusted average (standard error, SE) of job stressor scores by occupational class.*

Gender/Occupational class	n	Job demands		Job control		Supervisor support		Coworker support		Job insecurity	
				
		Crude average (SD)	Multivariate- adjusted average (SE)	Crude average (SD)	Multivariate- adjusted average (SE)	Crude average (SD)	Multivariate- adjusted average (SE)	Crude average (SD)	Multivariate- adjusted average (SE)	Crude average (SD)	Multivariate- adjusted average (SE)
	men										
managers	2,762	34.0 (5.2)	33.3 (0.2)	74.6 (9.0)	72.4 (0.3)	11.2 (2.0)	10.2 (0.8)	11.5 (1.3)	11.5 (0.1)	6.4 (1.5)	6.5 (0.1)
professionals	2,547	33.6 (5.2)	32.5 (0.2)	72.1 (8.7)	71.1 (0.3)	10.8 (2.1)	10.9 (0.3)	11.2 (1.6)	11.3 (0.1)	6.6 (1.6)	6.7 (0.1)
technicians	2,370	33.4 (5.1)	32.6 (0.2)	69.4 (8.9)	68.6 (0.3)	10.8 (2.2)	11.3 (0.3)	11.2 (1.6)	11.3 (0.0)	6.6 (1.7)	6.7 (0.1)
clerks	1,371	32.2 (5.3)	31.7 (0.2)	67.6 (8.8)	66.9 (0.3)	10.8 (2.2)	10.5 (0.2)	11.0 (1.7)	11.1 (0.1)	6.5 (1.5)	6.6 (0.1)
service & sales workers	230	32.1 (5.9)	31.9 (0.3)	65.9 (12.4)	65.0 (0.7)	10.9 (2.4)	10.8 (0.3)	11.1 (1.8)	11.2 (0.1)	6.3 (1.5)	6.5 (0.1)
craft and related trade workers	1,864	31.7 (4.9)	31.9 (0.2)	66.0 (10.5)	65.8 (0.3)	10.8 (2.2)	10.7 (0.3)	11.2 (1.6)	11.3 (0.1)	6.8 (1.9)	6.9 (0.1)
machine operators and assemblers	4,921	32.0 (5.0)	32.0 (0.1)	61.1 (10.5)	61.3 (0.3)	10.6 (2.2)	10.0 (0.2)	11.1 (1.7)	11.2 (0.0)	6.9 (1.9)	6.9 (0.0)
laborers	379	31.8 (5.5)	32.3 (0.3)	60.2 (10.8)	60.0 (0.5)	10.4 (2.3)	10.1 (0.3)	10.9 (1.8)	11.0 (0.1)	7.0 (2.0)	7.1 (0.1)

p for difference (df=7)		p<0.001	p<0.001	p<0.001	p<0.001	p<0.001	p<0.001	p<0.001	p<0.001	p<0.001	p<0.001
p for trend		p<0.001	p<0.001	p<0.001	p<0.001	p<0.001	p<0.001	p<0.001	p<0.001	p<0.001	p<0.001
	women										
managers	9	32.6 (7.0)	33.8 (1.8)	69.3 (11.9)	68.9 (3.3)	10.4 (3.2)	10.2 (0.8)	11.6 (2.6)	11.5 (0.6)	6.6 (1.6)	6.8 (0.7)
professionals	97	31.6 (4.7)	32.5 (0.7)	69.3 (8.2)	68.1 (1.3)	11.0 (2.4)	10.9 (0.3)	11.5 (1.7)	11.4 (0.2)	6.3 (1.3)	6.2 (0.3)
technicians	153	30.6 (4.8)	31.4 (0.6)	63.9 (9.1)	62.6 (1.2)	11.4 (2.1)	11.3 (0.3)	11.3 (1.5)	11.2 (0.2)	6.2 (1.4)	6.1 (0.2)
clerks	1,013	31.1 (5.6)	32.1 (0.5)	61.5 (9.2)	60.4 (1.0)	10.7 (2.3)	10.5 (0.2)	11.1 (1.7)	11.0 (0.2)	6.5 (1.5)	6.4 (0.2)
service & sales workers	102	33.4 (4.9)	34.2 (0.7)	57.0 (10.6)	53.8 (1.4)	11.1 (1.9)	10.8 (0.3)	11.5 (1.6)	11.5 (0.2)	6.2 (1.5)	5.9 (0.3)
craft and related trade workers	155	30.8 (4.3)	31.6 (0.7)	59.7 (9.4)	57.3 (1.2)	10.9 (2.2)	10.7 (0.3)	11.1 (1.5)	11.2 (0.2)	7.2 (2.2)	6.9 (0.2)
machine operators and assemblers	1,248	31.7 (4.9)	32.5 (0.5)	55.6 (10.5)	53.4 (1.0)	10.2 (2.4)	10.0 (0.2)	10.9 (1.6)	10.9 (0.2)	7.5 (2.3)	7.3 (0.2)
laborers	301	31.5 (5.2)	32.2 (0.6)	57.8 (9.6)	55.1 (1.1)	10.3 (2.6)	10.1 (0.3)	10.8 (2.0)	10.8 (0.2)	7.3 (2.2)	7.0 (0.2)

p for difference (df=7)		p<0.001	p=0.001	p<0.001	p<0.001	p<0.001	p<0.001	p<0.001	p=0.001	p<0.001	p<0.001
p for trend		p=0.015	p=0.346	p<0.001	p<0.001	p<0.001	p<0.001	p<0.001	p=0.001	p<0.001	p<0.001

*: Multivariate-adjusted average was estimated by ANOVA controlling for study site, age group, education, marital status, chronic medical condition, and personality traits (neuroticism and extraversion)

The proportion of those in the high job strain group was significantly different among occupations in men (p<0.001), with a greater proportion in low-class occupations (Table 3). Men in low-class occupations showed a significantly greater prevalence OR of being in the high job strain group. A similar pattern was observed for women, while female service workers and machine operators had a higher prevalence OR of having high job strain.

**Table 3.**
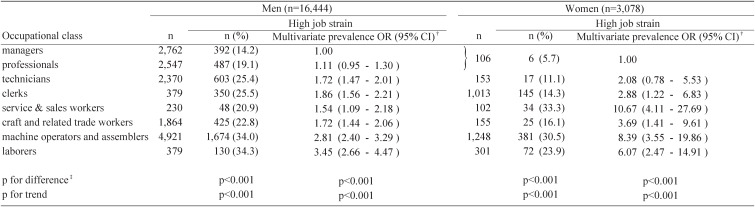
Associations between occupational class and high job strain* as the combination of high job demands and low job control: crude proportions and estimated prevalence odds ratios (ORs) with 95% confidence intervals (CIs).

* : A total of 4,109 (25.0%) of men and 680 (22.1%) of women were in the high job strain category.

† : Multiple logistic regression controlling for survey site, age group, education, marital status, chronic medical condition, and personality traits (neuroticism and extraversion).

‡ : Df=7 for men and df=6 for women.

## DISCUSSION

The present study revealed a clear occupational class gradient in exposure to low job control and high job strain among employed men and women in Japan with a greater risk of exposure to low job control and high job strain in low-class occupations. This pattern is consistent with previous findings of the United States Quality of Employment Survey^2^ and the Whitehall II study in the United Kingdom^1^ and with previous findings in Japan.^6^^-^^8^^,^^10^ The present study showed that about 12-13% of the variance in the job control score was explained by occupation and that less than 1% of that in the job demands score was explained by occupations among men or women. On the other hand, a total of 14% and 1% of the variance in job control and job demands, respectively, were explained by occupations classified by the same method (using the first-digit ISCO88 coding) in the JACE study in European countries.^19^ The association between occupational class gradient and job strain or job control seems equal to or only slightly smaller in Japan than that in Western countries.

The observed occupational class gradient of job strain does not fully explain a previous observation of greater coronary heart disease risk factors^4^ among managers in Japan. Job strain has been shown to be associated with fewer leisure-time physical activities in previous studies in Japan.^20^^,^^21^ However, using the same sample, we previously reported fewer leisure-time physical activities both in high-class occupations and low-class occupations.^5^ This apparent discrepancy may be attributable to other work-related factors, such as greater job commitment among managers.^5^ Such work-related factors other than job strain, associated with deteriorated health behaviors, such as fewer leisure-time physical activities, may attenuate the occupational class gradient of health due to job strain in Japanese workers.

Women in our sample had a greater prevalence OR of high job strain associated with being in low-class occupations than men. In addition, job insecurity was greater in low-class occupations among women workers. Recent studies have shown that job insecurity is also associated with poor health.^22^^,^^23^ Females are frequently employed in support roles.^20^ Furthermore, they are assumed to have more job mobility than men in Japan because they bear a larger share of childcare responsibilities that men do and might have to change or give up jobs as a result. Women in low-skill occupations may feel that their positions are often unstable. The occupational class difference in job strain and job insecurity may be greater among women than among men in Japan. The finding is inconsistent with previous one that there was a less clear employment grade difference in health status among women in Japan.^3^ A possible explanation for this discrepancy is that women’s health is affected or moderated by a number of other factors than job strain, such as family responsibilities and social relationships outside of work. The social class and occupational class of their spouses, rather than their own occupational class, may be more important for women’s health when a woman is economically dependent on her spouse. Besides the occupational class gradient, job strain was greater for service and sales workers among women because of the greater job demands in this particular occupational group. In addition to occupational class, job strain might arise from a situation specific for each occupation, such as increased workload among female service and sales workers.

Differences in the scores of supervisor and coworker support were small for men and women, while the scores were greater in high-class occupations. This is consistent with previous observations among men^6^^,^^8^ and women^10^ in Japan. The gradient in social support at work along with employment grade was not clear among women in the White-Hall II study^1^ or in other studies in the Unite States.^24^ Occupational class may not be a strong determinant of worksite social support in Japan or in Western countries.

Our sample consisted of only full-time workers from relatively large companies. It is not clear to what extent the present findings can be generalized to other company employees, part-time workers and employees in small enterprises. Caution should also be exercised because the survey was completed during a severe and sustained economic recession. Despite a large number of total respondents, the number of women in high-class occupations was still relatively small. The findings should be replicated in a community-based survey or a survey with a special focus on women in high-class occupations.

An internationally standardized measure of classification of occupations was used. However, as noted previously, it was difficult to code the occupations based exclusively on a respondent’s description on his/her job title and responsibility. The occupational coding may not be reliable and may differ among raters, resulting in a possible underestimation of the association between occupational class and job stressors. In addition, there are several ways to determine occupation-related social class. While we employed an indicator of occupational class based on the skills and training required for occupations, the employment grade according to salary was used in the White-Hall II study.^1^ These occupational class indicators may have a different implication. The use of multiple occupational class indicators should be encouraged in future research.

An internationally standardized measure of job stressors, i.e., the JCQ was used. The reliability and validity of the JCQ scales used here have been tested and found at acceptable levels for most scales. However, the low reliability of the job insecurity scale may result in an underestimation of the association between occupational class and exposure to job insecurity. Other measures of a similar construct with a better reliability should be used to replicate the findings, such as a job future ambiguity scale.
